# The New Anæsthetic—Keroselene

**Published:** 1861-11

**Authors:** 


The new Anæsthetic—Keroselene.—We have before us a phial of liquid, colorless like water, and possessing a strong permeating smell not unlike chloroform, but with other qualities peculiar to itself. This liquid is *Keroselene*, and as it is at present attracting considerable attention in the medical world as an anæsthetic agent, we can not do better than give our readers some account of it. Keroselene is a volatile product, arising from the final distillation of petroleum, or coal-oil; chemically it is a pure liquid, hydro-carbon, its specific gravity being lighter than that of any other known liquid. The peculiar properties of this liquid were first observed in the manufacture of kerosene oil; as the men engaged in the department where the keroselene was formed, were frequently found in a partially anæsthetic condition, and similarly affected to persons under the influence of "*laughing gas.*" The gentleman to whom we owe the discovery of a mode of purifying keroselene to such a point as to render it a useful anæsthetic, is Mr. Joshua Merrill, superintendent of the "Downer Kerosene Oil Co.," Boston, Mass. Mr. Merrill presented a specimen of this liquid to the "Boston Society for Medical Improvement," when on a cursory examination, Dr. H. J. Bigelow pronounced it in his opinion "as strong at least as ether, tasteless as water, while its vapor is no way irritating. On the contrary, its flavor is agreeable, and resembles a dilute chloroform, with a whiff of coal-tar or creosote. It is also abundant and cheap (one dollar per gallon.) If this is an anæsthetic of the character it seems to be, at once effectual, agreeable, tasteless, without subsequent flavor or odor, it will supersede ether; and it has certainly at this moment a remarkable air of promise." The new agent was then referred to the hospital surgeons for experiment, and on motion, Dr. Bigelow was requested to draw up a report of the results of their investigations. In the *New York Medical Times*, Dr. Cutler, of Woburn, Mass., related some experiments made with keroselene, which were completely successful. It had also been used externally in neuralgia. Dr. Bigelow, of Boston, afterward experimented upon himself and upon various surgical cases, and proved its anæsthetic properties, though in one or two instances there was tendency to asphyxia and convulsions. There is but little doubt, however, that when prepared pure, this will prove an invaluable anæsthetic.—*N. Y. Dental Journal.*

